# Lévy like patterns in the small-scale movements of marsupials in an unfamiliar and risky environment

**DOI:** 10.1038/s41598-019-39045-0

**Published:** 2019-02-25

**Authors:** B. Ríos-Uzeda, E. Brigatti, M. V. Vieira

**Affiliations:** 1 Laboratório de Vertebrados, Instituto de Biologia, Universidade Federal do Rio de Janeiro, Caixa Postal 68020, 21941-590 Rio de Janeiro, RJ Brazil; 2 Instituto de Física, Universidade Federal do Rio de Janeiro, Av. Athos da Silveira Ramos, 149, Cidade Universitária, 21941-972 Rio de Janeiro, RJ Brazil

## Abstract

We investigate the movement patterns of three different Neotropical marsupials in an unfamiliar and risky environment. Animals are released in a matrix from which they try to reach a patch of forest. Their movements, performed on a small spacial scale, are best approximated by Lévy flights. Patterns of oriented and non-oriented individuals - with forest patches within or beyond their perceptual range - differ only slightly in the value of their exponents. These facts suggest that, for these species, the appearance of Lévy flights is the product of animals innate behaviour that emerges spontaneously, as a neutral characteristic proper of a default movement mode for alerted animals.

## Introduction

Loss and fragmentation of natural habitats affects ecological processes such as species interactions, trophic dynamics and dispersal processes, and can cause declines in population density, changes in species richness and in community composition^1–3^. Habitat loss and fragmentation also affects movements, making it difficult for an animal to move in unfamiliar enviroments, such as the matrix between fragments and, to reach new suitable habitats^4,5^. In the Neotropics, marsupials are an example of small non-flying vertebrates affected by fragmentation^3^. Most species of Neotropical marsupials are forest species, inhabit the forest and use the matrix just to move between patches. Although predation risk is likely higher in the matrix, within the forest they are also susceptible to be predated by a wide range of animals, e.g. snakes or raptors^6^. For this reason, in both forest and matrix specific patterns of movement are important to minimise predation risk, to increase survival probabilities^7^, and the success in reaching a forest patch.

In movement ecology, the description of animal movement can be based on the characterisation of different stages that can be related to three questions: when to move, where to move, and how to move^8,9^. When to move involves factors related with evaluation of food, shelter, or reproduction availability. Where to move is generally determined by the previous knowledge of the environment, perceptual capacity, and ability of orientation. How to move, is related to locomotion ability, and to numerous internal and external factors that can provide assistance to movements, improving the chance of reaching the final location.

A first step in the study of animal movement patterns is the characterisation of individual paths measuring their turning angles (the difference between previous and current direction of motion), and their step lengths (the distance between the turning points)^10–14^. This type of analysis has been frequently carried out for trajectories in two dimensions (see for example^15,16^), and sometimes also in three dimensions^17^. A more comprehensive description of movement patterns should also consider the temporal dynamics of these trajectories, characterising the movements by means of the spatial and temporal distributions of animal positions. This approach, in analogy with classical models of diffusion of particles, allows to explore the mathematical and statistical properties of these distributions, and to connect them with the ecological processes underlying these movements^13,18–20^.

Historically, a first tentative description of animal movement patterns introduced the use of a simple random motion, generally described by means of a Brownian motion. In a second moment, a new approach was introduced with the use of correlated random walks, a probabilistic model where movements present some type of memory of the previous motion^10^. Correlated random walks were introduced with the aim of teasing apart searching and foraging from random movement behaviour, and they can be considered a milestone in the development of movement ecology discipline^9^. Later, an alternative description of animal movements was proposed with the introduction of Lévy processes^21,22^. This approach originated in the field of statistical mechanics, and nowadays presents large application in physics and natural sciences^23^. It presents characteristic trajectories marked by abrupt long jumps which connects clusters of frequent short displacements, generating a characteristic scale-invariant structure and a possible super-diffusive behaviour^20,24^. Since the end of the 90’s this process has attracted the attention of the ecologists for being considered a result of optimal foraging theory^11,25^, and a large amount of studies, connected with its use for describing searching and foraging movements, has flourished since then (e.g. Viswanathan *et al*.^11,25–29^, Bartumeus *et al*.^20,30,31^, Bartumeus^32,33^, Edwards^12^, Edwards *et al*.^34,35^, and Reynolds^16,19,36–38^).

Controversy around this approach emerged, questioning the results of many early studies, generally because of the use of inappropriate statistical techniques^12,34,35^. However, in the last ten years, many studies confirmed that Lévy flights are a good approximation to the patterns of movement of different species, either in the case of foraging animals looking for scarce preys, or emerging from a general innate behaviour^16,19^. However, cases of Lévy processes in nature involved movements of individual organisms over large areas, during many days or periods of activity. Also, animals were engaged in foraging activities, whether in a familiar environment or not.

The implementation of the three previous movement models (Brownian motion, CRW, and Lévy processes) in terms of discrete models is simple. The common idea is to assume that the movement of an animal consists in a discrete series of steps separated by events of reorientation^12,25,30^. The step-length *l* is selected from a distribution *P*(*l*) and its direction, defined as the turning angle in relation to the the direction of the previous movement, is picked up from a distribution *P*(*θ*). The Brownian random walk is characterized by a *P*(*l*) with a finite variance. Generally it corresponds to a Gaussian but even a fixed step length is a possible choice. The direction of every step is isotropic, with *P*(*θ*) represented by a uniform random distribution. The correlated random walk presents *P*(*l*) with a finite variance. However, the directions of the movements are not isotropic, introducing a correlation between the directions of subsequent movements. In fact, the persistence in the direction (the degree of correlation of the random walk) is controlled by the shape of the turning angles distribution^10,39^. Generally, this effect can be obtained using a *P*(*θ*) following a wrapped Gaussian distribution.

In the case of the Lévy processes, usually called Lévy flights, step-lengths have a probability distribution that is heavy-tailed: $P(l)\propto {l}^{-\mu }$, with 1 < *μ* ≤ 3, and the directions are isotropic^25,40^. Note that *μ* > 3 generates trajectories with large-scale properties typical of a Brownian motion, whereas, approaching *μ* = 1, ballistic trajectories are recovered. Lévy flights have been suggested to be the best solution for solving the problem of random search^11,41^. In particular, Viswanathan *et al*.^25^ proved that in environments where the resources are homogeneous and scarce, if search targets are not depleted or rejected once visited, but can instead be profitably revisited, the optimal *μ* is 2. This result generalises to *μ* ≈ 2 in fragmented landscapes^42^.

The main purpose of this study is to characterise the movement patterns of three Neotropical marsupials released in a matrix of an unfamiliar habitat. The three species represent the range of body sizes of Neotropical marsupials that are generalist, mostly omnivores, and are capable of climbing but mostly use the forest floor or understory. The matrix in the study areas was composed of pastures, which represents an unfavourable and risky habitat for these animals. For this reason, we can consider that it induces animals to keep alert of potential predations and to seek for a safe place, which, in our experimental set-up, is a patch of forest at a given distance. This is an important difference in relation to the majority of the existent literature, which has reported results related to movements of animals searching for food. Simulations suggest that predation risk may favour smaller values of the *μ* exponent of Lévy walks^43,44^, but may also favour large values of *μ* depending on the foraging strategy of the predator^43^. Another relevant novelty is that our study is developed on spatial and temporal scales smaller than the ones usually considered in previous studies with larger animals. The trajectories of the small marsupials studied here were performed within a small region of the matrix, and represent only part of one cycle of activity of an individual (less than one day).

Finally, two distinct situations were considered: animals that are able to orient themselves in the direction of the patch, and animals that are not able to orient themselves. In this way, we are able to unfold a rich comparison between the movements of three species representing a range of body sizes, both for oriented and non-oriented animals.

## Material and Methods

### Study site and field methods

The dataset is the result of a series of studies performed from 2007 to 2010 to determine the perceptual ranges and movement behavior of marsupials in an Atlantic Forest landscape in Rio de Janeiro state, Brazil. The field work was conducted in the Guapi-Macacu river basin (22°25′S and 42°44′W), which is part of the municipality of Guapimirim and Cachoeiras de Macacu. The climate is mild humid-mesotermic^45^, and vegetation of the region is classified as dense evergreen forest (“Ombrófila Densa”^46^). Vegetation of the fragments is disturbed to various degrees, with a relatively open understory and canopy, and is characterized by the presence of palms (*Astrocaryum aculeatissimum*), *Cecropia sp*., and lianas^47^. The region of the Guapi-Macacu River basin has a long history of human occupation, and currently the landscape is characterized mainly by small forest fragments (<200 ha) structurally isolated by a matrix of urban areas, pastures, plantations, and paved roads^3,48,49^. The Guapi-Macacu river basin has ca. 45% of forest cover, part of it old-growth forest at the base and on the slopes of sierras, along the northern portion of the river basin^50^. The matrix where forest fragments are inserted is heterogeneous, with different plantations and pasture systems^48,49,51^.

Perceptual ranges were determined by releasing individuals in the matrix at different distances (30, 50, 100 and 200 m) from an unfamiliar forest patch. The three species studied are *Didelphis aurita*, *Marmosa paraguayana* and *Philander frenatus* and they represent the range of body sizes of Neotropical marsupials (mean body mass of individuals released: 850 g in *D*. *aurita*, 395 g in *P*. *frenatus*, and 115 g in *M*. *paraguayana*). In addition to the tracking of trajectories, the experimental set-up allowed to estimate the ability of animals to orient themselves in the direction of a forest patch and to classify the data on the bases of this characteristic.

As in^52^, it was assumed that animals, being released in an open and unfamiliar habitat, would look for refuge which would corresponded to the nearest patch of forest. All animals were released just once to avoid possible cumulative experiences, and the site of release was distant, at least, 1000 m from the point of capture to reduce the possibility of familiarity with the region.

**Figure 1:**
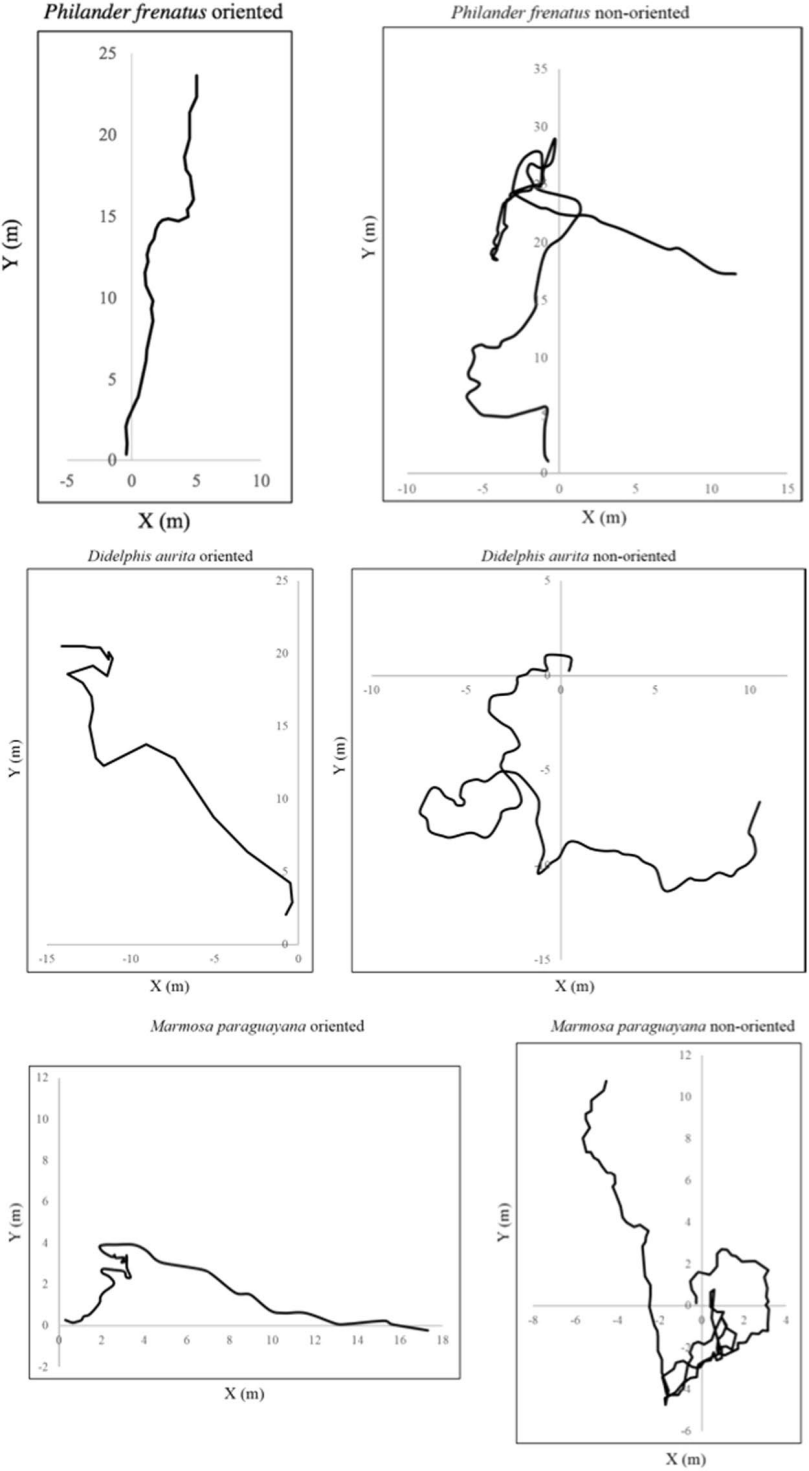
Experimental trajectories. Examples of the trajectories of oriented and non-oriented individuals of the three species of marsupials. Oriented individuals of *P*. *frenatus* and *M*. *paraguayana* were released at 100 m from the nearest forest fragment, 200 m in *D*. *aurita*; non-oriented *P*. *frenatus* and *M*. *paraguayana* were released at 200 m from the nearest forest fragment, 300 m in *D*. *aurita*. Note that, at the scale of these figures, the body sizes of these animals are of the order of a point. The data-set variability, even inside each class, is very hight.

Individuals were equipped with a spool-and-line device, that consists of bobbinless cocoons of nylon thread wrapped in a polyvinyl chloride film^53–55^. Each device weighs 4.8 *g* and the thread is 480 *m* long^56^. Devices were attached to the fur between the shoulders of each individual by using an ester-cyanoacrylate-based glue (Henkel Loctite Adesivos Ltda., Manaus, Brazil). The thread released by the device allowed mapping the animals’ path (see Fig. 1). The direction of the path was measured aligning a compass with the direction of the line to the next point of change of direction greater than five degrees (as in^52^, but without the rediscretisation to 10 degrees in^57–59^), and the linear distance between points was measured with a tapeline. The experiment ended when the animal reached the target or it went out of the matrix^57^. Paths were followed up to 170 m, to allow more individuals to be tracked. Occasionally the experiment ended before 170 m of path, when the animal reached the target^59^. Trapping and handling conformed to guidelines sanctioned by the American Society of Mammalogists^60^. This study was approved by IBAMA/MMA (Authorization numbers 87/05-RJ, 099/06-RJ, 13861-1, 13861-2, 16703).

Trajectories were classified as belonging to an oriented or a non-oriented animal on the basis of the estimated perceptual range of these species, defined as the ability to perceive a forest patch at a given distance from the release point. The estimation of the perceptual range was obtained in^52,58^ evaluating the mean vector of orientation of the first 20 m of path which was used to determine if individuals released at a certain distance were significantly oriented towards the nearest forest patch. Distances to a forest patch beyond perceptual range where the ones with non-significant concentration of angles in the direction of the closest forest patch. Perceptual ranges were estimated as 100 *m* for *M*. *paraguaiana* and *P*. *frenatus*, and 200 *m* for *D*. *aurita*^52^. Based on these results, we considered as oriented the animals released at a distance smaller or equal to the perceptual range, and non-oriented the other cases.

### Statistical modeling and parameter estimation

The principal aim of our analysis is to evaluate the distribution of the step-lengths *l*, defined as the distance between two successive turning points. If the measured trajectories are directly used to evaluate *l*, the turning points are simply selected as all the points with an angle larger than a minimal angle *θ*_*min*_. For our data set it would be natural to choose *θ*_*min*_ = 5. However, this ad-hoc discretization of the movement patterns would be arbitrary and the estimated distribution *P*(*l*) would strongly depend on this choice^17^. We overcame this problem with a recent approach introduced in^40^, which analyse not the trajectories, but their projections. In analogy with that procedure, a trajectory in 2D is projected on one axis, the *x*-axis for example. A projected step *l*_*x*_ is defined as the distance between two inversions in the direction of the movement of the projected trajectory. Similarly, we obtain the step-lengths along the *y*-axis (*l*_*y*_). It was analytically proven that if the distribution of steps in the original 2D path followed a power law, the distribution of the projected paths preserves the same power-law relationship^40^. In the case of an exponential distribution, the projected data do not preserve the same functional form, but they maintain the general exponentially decaying behaviour. Following this method we were able to investigate the shape of the *P*(*l*) distribution using a segmentation procedure that does not depend on any arbitrary choice. Moreover, any correlation between turning angles in the original dataset would be eliminated in the projected data. As pointed out in^17,40^, the operation of projection, however, causes the proliferation of spurious data corresponding to projected steps smaller than the minimum 2D step-length present in the original dataset. These data must be excluded from the final analysis. Moreover, as the last measured step-length can have a length influenced by the ending of the spool, they were eliminated from the data set. It follows that also rare events, with a unique straight trajectory were excluded. In this way, pure ballistic movements are ruled out. Finally, after having checked that the two distributions of *P*(*l*_*x*_) and *P*(*l*_*y*_), as expected, do not show any discrepancy, we combined in *P*(*l*) the data coming from the two projections.

Once obtained *P*(*l*), we face the problem of estimating the best model that describes it. The characterisation of a power-law behaviour could be obtained using a simple Pareto distribution: *f*(*x*) = (*μ* − 1)*a*^*μ*−1^*x*^−*μ*^, for *x* ≥ *a*. However, for our dataset this distribution should be ruled out because surely important truncation effects are present and a limit to the maximum *l* value must be taken into account. A realistic animal movement is characterised by physiological and circadian rhythms that cause direct limits on the maximum step length. Second, and more important, inevitable truncation effects come from the measurement process, which cannot measure step-lengths larger than the length of the spool. For these reasons, it is more appropriate the use of a truncated Pareto distribution, defined as: *g*(*x*) = (1 − *μ*)/(*b*^1−*μ*^ − *a*^1−*μ*^)*x*^−*μ*^, for *a* ≤ *x* ≤ *b*. Although for reasonable high values of the *μ* exponent the use of the Pareto or truncated Pareto distribution can lead to comparable results, for small values of *μ* (typically *μ* < 2) the difference in the estimation is substantial, as can be proven by numerical tests, and the use of the Pareto truncated distribution is essential.

The best estimation of the parameters of this distribution was obtained using the Maximum Likelihood Estimation (MLE), which is more reliable compared to classical least squares methods (see, for example^61,62^, for a detailed discussion). For a truncated Pareto distribution, the estimation of the *μ* parameter is given by the numerical solution of the equation^62,63^:

1 $$\tfrac{1}{n}\,\sum _{i=1}^{n}\,{ln}({l}_{i})=\tfrac{1}{\mu -1}+\tfrac{{b}^{1-\mu }\,{ln}(b)-{a}^{1-\mu }\,{ln}(a)}{{b}^{1-\mu }-{a}^{1-\mu }}$$

where *a* = *min*(*l*_*i*_) and *b* = *max*(*l*_*i*_).

Unfortunately, in contrast to the Pareto distribution, there is no simple tests to determine whether a truncated Pareto model generates an appropriate goodness of fit^63^. For this reason, the estimation must be supplemented by a graphical check of the data. The inspection was realised comparing the best fit model to the frequency of the step-lengths of the projected movements using a log-binned histogram. Survival functions were not introduced because, for small values of *μ*, it is not possible to make a clear comparison^40^.

A model selection approach was used to compare models based on the whole dataset, considering all step-lengths. To quantify the evidence supporting each model we used the Akaike information criterion (*AIC*), which compares models likelihoods penalizing models with more parameters^64^. The *AIC* is calculated as follows: *AIC* = 2 *K* − 2 *L*, where *L* is the maximum log-likelihood and *K* is the number of parameters of the model. The maximum log-likelihood was estimated following Edwards^35^. The model with the lowest Akaike information is the best supported model. In addition to the Pareto and the truncated Pareto distribution, we consider also the exponential distribution: *h*(*x*) = *λ* exp(*λa*) exp(−*λx*) for *x* ≥ *a*.

The model selection approach was also used to compare support between exponential and truncated Pareto for different subsets of the data. This approach can be useful for detecting the possible existence of some typical scale, corresponding to the occurence of an exponential truncation. This analysis was obtained performing a sequential maximum likelihood estimation of model parameters and a model selection on different subsets of our data. A given subset *n* was defined from the previous *n* − 1 set by subtracting its smallest value. Looking at the *AIC* weights for each subset, plotted as a function of its smallest value, this procedure can tell us if a critical step value exists where there is more support for an exponential rather than a truncated Pareto distribution.

After having analysed the projected data, we also inspected the behaviour of the original empirical data, estimating the corresponding distributions *P*(*l*) and *P*(*θ*), and we implement a simulation based on a discrete walk, for synthetically reproducing the empirical data-set. Our aim is to test the robustness of our analysis and to clarify the relationships linking the original and the projected data. For this part of the study we focus our attention on the data of oriented individuals of *Philander frenatus* because these data are abundant and present well behaved distributions.

Most of the data generated and analysed during the current study are included in this article and its Supplementary Information. The rest of the raw data are available from the corresponding authors upon request.

## Results

**Figure 2:**
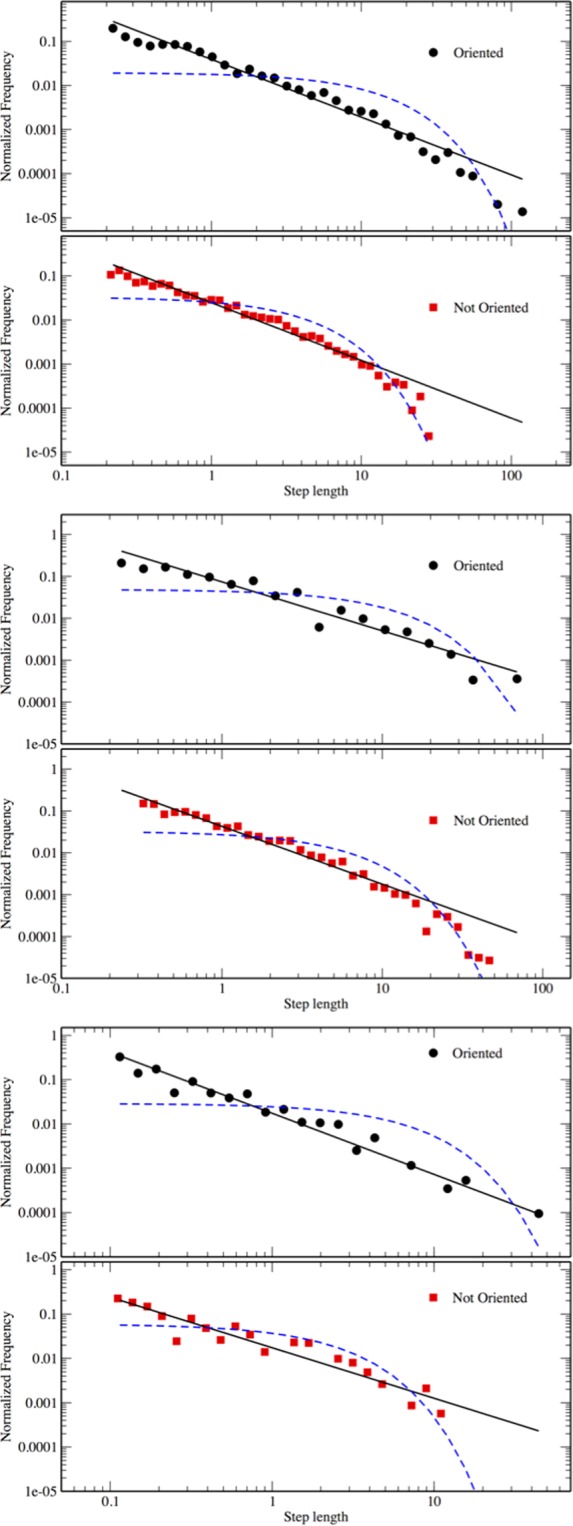
Step-lengths distributions. Log-binned data with the best estimated Pareto-truncated (continuous line) and exponential distribution (dashed line). Step-lengths are expressed in meters. From top to bottom: *Philander frenatus*, *Didelphis aurita*, *Marmosa paraguayana*. Black dots are data from oriented animals, red squares from non-oriented ones.

The Pareto-truncated distribution always presents the lowest *AIC* values considering the whole dataset (see Table 1). Support for the Pareto-truncated is much higher than the competing distributions, as indicated by the Δ*AIC* values, which, in the worst case, is as hight as 28, and the Akaike weights, which suggest that the distributions cannot easily be mistaken for Pareto or exponential distributions (see Supplementary Information for further details on the model selection). A visual inspection is sufficient to persuade that the fitting of the exponential to our dataset is very poor (see Fig. 2). Table 2 resumes the results of this analysis for the different species, distinguishing between oriented and non-oriented animals.

**Table 1:** The Akaike information criterion values (*AIC*) for the Pareto truncated, Pareto and exponential distributions.

Species		Pareto-truncated	Pareto	Exponential
*Philander frenatus*	Oriented	2529	2727	3522
	Non-oriented	4458	4951	5246
*Didelphis aurita*	Oriented	992	1080	1236
	Non-oriented	3249	3662	3755
*Marmosa paraguayana*	Oriented	227	255	465
	Non-oriented	179	215	209

Further results are given in the Supplementary Information.

**Table 2:** Values of *μ* for the Pareto truncated distribution for the three species of marsupials.

Species		Observations	Sample Size	*μ*	*a* (m)	*b* (m)
*Philander frenatus*	Oriented	42	541	1.31 ± 0.03	0.20	128.76
	Non-oriented	57	1161	1.38 ± 0.02	0.22	59.46
*Didelphis aurita*	Oriented	19	182	1.17 ± 0.05	0.23	79.63
	Non-oriented	41	875	1.38 ± 0.02	0.22	48.82
*Marmosa paraguayana*	Oriented	6	88	1.38 ± 0.07	0.10	40.03
	Non-oriented	3	65	1.14 ± 0.09	0.10	11.20

Observations indicate the number of trajectories analysed. Each trajectory is the whole track of a single individual. Sample size corresponds to the number of regressed data after eliminating the spurious *l* smaller than the minimal step of the original (not projected) data. The values of parameters *a* and *b* are expressed in meters.

**Figure 3:**
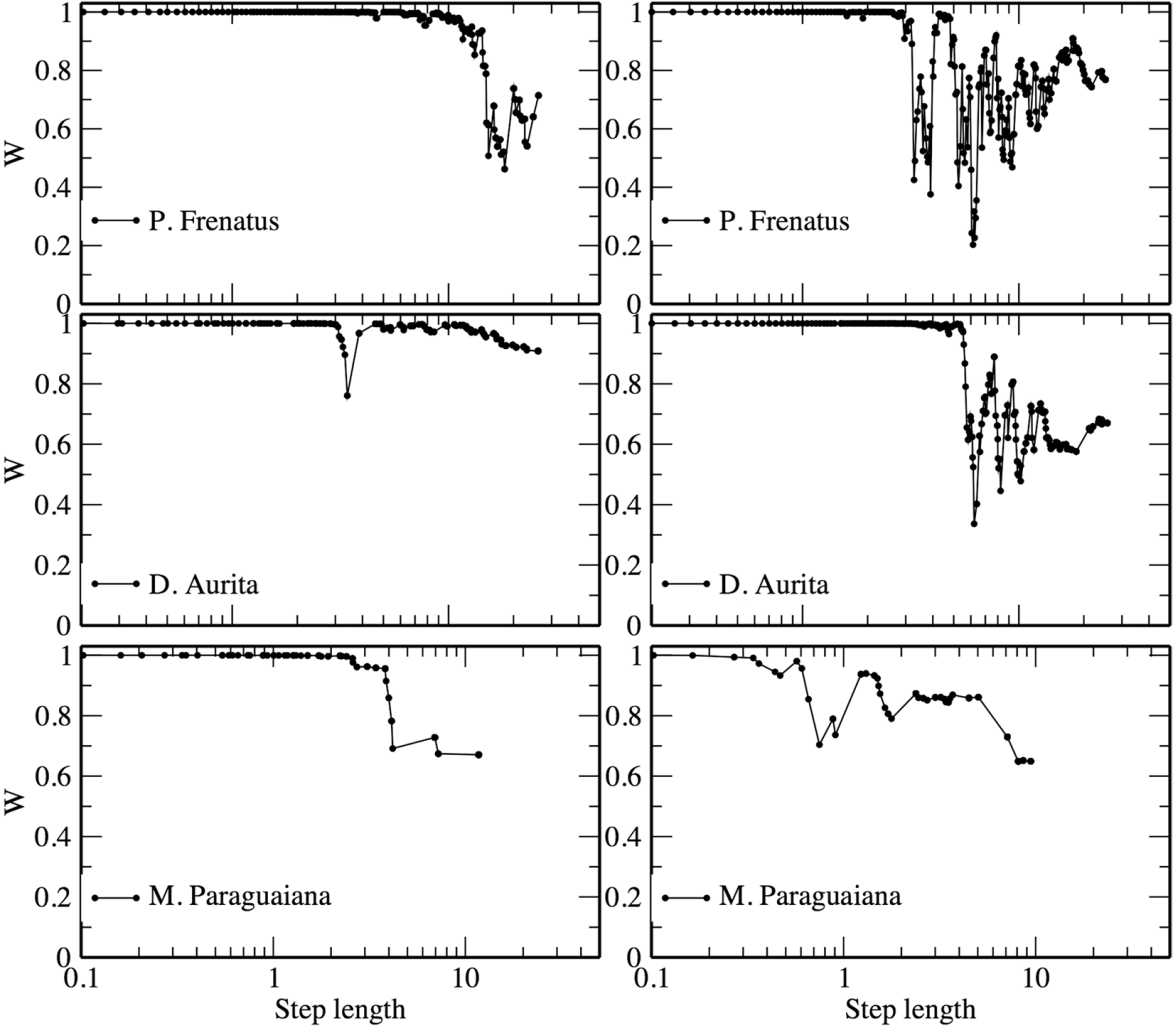
The sequential maximum likelihood estimation. Weight of evidence (Akaike weights) favouring Pareto-truncated compared to exponential distribution along different subsets of the step lengths, for oriented (left) and non-oriented (right) individuals. Akaike weights were estimated by sequential maximum likelihood, and displayed as a function of the smallest step length in each subset. A weight of one corresponds to the maximum weight of evidence in favor of the Pareto-truncated distribution.

Based on the sequential maximum likelihood estimation on different subsets of our data, only non-oriented *D*. *Aurita* and *P*. *Frenatus* present a large step value for which the weight of evidence in favor of the exponential model grows, but rarely passing the critical 0.5 value (see Fig. 3). In all the other cases the power-law model is the most probable one at all scales, even if there is a general deterioration in the evidence. Therefore, the inference of power law behaviour is robust, and there is no evidence of best exponential fit on subsets of the data.

**Figure 4:**
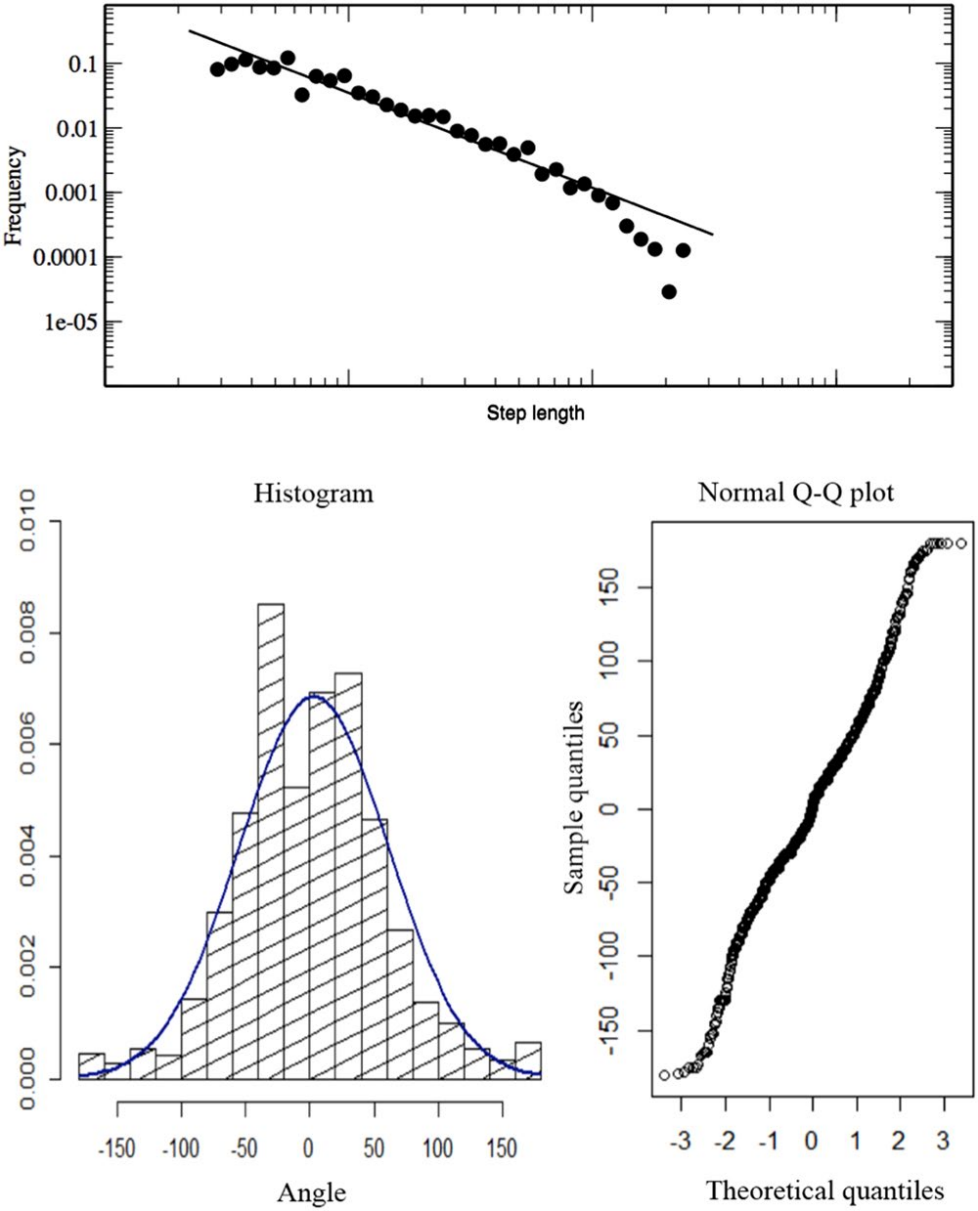
Original empirical data. Top: Distribution of step lenghts of the original empirical data for oriented *Philander frenatus*. Step lengths are log-binned. The continuous line represents the best estimated Pareto-truncated distribution. Bottom: Turning angle distribution of the same data. Normality of the data can be appreciate looking at the q-q-plot.

For the analysis of the original empirical data, step-lengths (*l*_*o*_) are defined as the distance between two turning points. This is equivalent to set the threshold of the turning angles to 5°. The inspection of the *P*(*l*_*o*_) distribution shows a behaviour well described by a truncated Pareto distribution. The estimation using the MLE gives an exponent of 1.47 ± 0.02 (see Fig. 4).

For performing the analysis of the angles at the turning points, we measure the net angular change in orientation between two consecutive directions along the animal path (*θ*). The corresponding *P*(*θ*) distribution can be well described by a Gaussian centred around zero and with a standard deviation of 58° (see Fig. 4). An analogous behaviour was detected for all the species, for oriented and non-oriented individuals (see Supplementary Information, Fig. S1).

**Figure 5:**
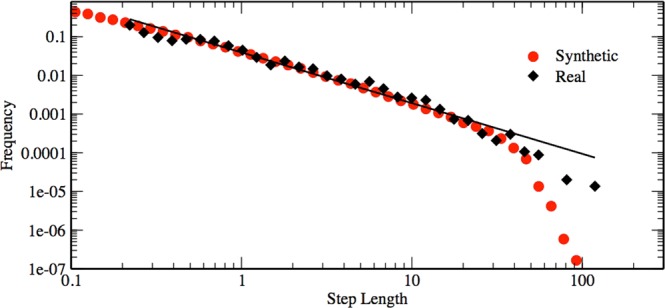
Simulated data. Comparison of the histogram of the projected step-lengths as obtained from the field data (black diamonds) and from the synthetic data (red dots) generated by our simulation. These last data show a clear exponential cutoff for large step sizes. This fact can be ascribed to the process of projection, as it is present also for simulations with Pareto truncated distribution with no correlations in the angles. The continuous line represents the best estimated Pareto-truncated distribution for the synthetic data.

Given the distribution *P*(*l*_*o*_) and *P*(*θ*), we are able to build up the simulation for synthetically reproducing the empirical data-set. This is obtained performing a discrete walk which implements, at the same time, both a Pareto truncated step-length distribution and a Normal distribution for the turning angles. We ran a long simulation generating a walk of 5 × 10^4^ steps. For this simulation we fixed the maximum value of the step-length to 48 m, and the minimal value equal to 0.01 m. This corresponds to consider an upper cutoff, as effectively measured from the field data, and a minimal one, to account for the necessary mathematical constraint of the distribution. The same analysis performed on the original field data was applied to this synthetic data-set: the simulated path is projected on the *x* and *y* axis to obtain the corresponding step-lengths for the projected data. As can be observed in Fig. 5, the synthetic projected data perfectly reproduce the behaviour of the real projected data, presenting an estimated *μ* value equal to 1.299 ± 0.003.

## Discussion

Small scale movements of the the three species of marsupials are best described by Lévy flights. The projection method, *MLE* and *AIC* model selection give strong support to Lévy flights as the best model compared to alternatives. The possible existence of a marked scale in terms of an exponential truncation is ruled out by the results of the sequential maximum likelihood estimation on different subsets of our data. The marginal deterioration of the support in favour of the truncated Pareto distribution can be interpreted considering the presence of measurement truncation effects and the use of the projection method for truncated Pareto distribution, as clearly shown in Fig. 5.

**Figure 6:**
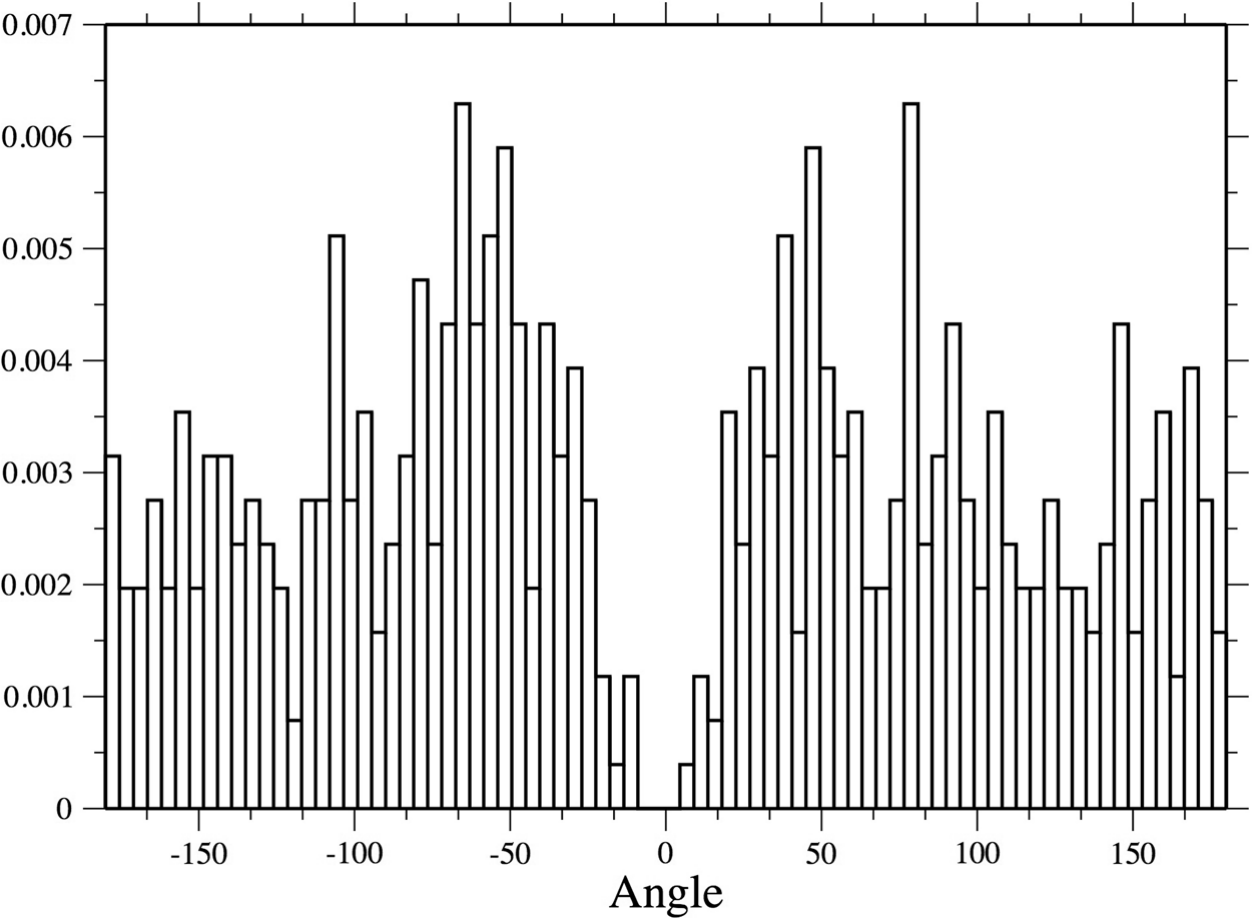
Turning angles. Distribution of the turning angles for oriented *Philander frenatus* when measured in correspondence of the points identified as turning points in one dimension, combining the angles in the x and y dimension. As can be appreciated, angles sufficiently far from zero are distributed approximately uniformly. The behaviour around zero is produced by the effect of coalescence of small turning angles during the projection operation and by the fact that the recorded changes of direction are always greater than five degrees. Similar results can be obtained for all the considered dataset.

These conclusions were reinforced by the outputs of the original data-set and the simulated data. The original data-set present a step distribution well described by a truncated Pareto distribution and a turning angle *P*(*θ*) distribution well approximated by a Gaussian one, similarly to the observed in the turning angles of wandering albatrosses^17^. This result is consonant with the fact that the motion of these animals presents a general directional persistence, which causes small turns centred around the previous direction. It is important to note that a genuine Lévy flight is defined with a *P*(*θ*) given by a random uniform distribution. However, we care for being able to describe the projected data as a standard Lévy flight and not the original empirical data, which present an arbitrary segmentation. In the case of projected data, the turns must be calculated only when they correspond to turning points (inversions) in one dimension. In this case, a uniform distribution was obtained, as can be observed in Fig. 6. Moreover, this projecting operation results in the coalescence of small angles, which coherently generates longer step-length, and it accounts for the directional persistence of the paths. For this reason, the *μ* value for the distribution of *P*(*l*_*o*_) is larger than the one obtained for the projected data. The results of the simulation corroborates these considerations: given a walk with a truncated Pareto distribution and a strong correlation in the direction of motion, the distribution of the projected steps presents a clear Pareto truncated distribution with a smaller exponent. In particular, the value of the exponent obtained with our simulation is coherent with the one extracted from the real data.

Our study is developed on spatial and temporal scales smaller than the ones usually considered in previous experiments with vertebrates^29,65,66^. In those experiments, animals were generally medium and large sized and they were treading long distances, with data usually corresponding to a discrete sample of the trajectory. In contrast, our study was realised with small vertebrates, in an area smaller than 0.25 *km*^2^, using a continuous tracking method which allows an accurate description of the trajectories shape. The fact that also in this condition Lévy flights are detected, reinforces the idea that these phenomena are characterized by a scale-free process, where the same behaviour can be recorded at different scales^25,32^.

The characterisation of the movement based on a Lévy flight is common to the three species of marsupials, even if they present different habits. In fact, *M*. *paraguaiana* is a more arboreal species, *P*. *frenatus* is generally terrestrial, and *D*. *aurita* presents both arboreal and terrestrial habits^67^. This result suggests that these patterns of movement present an interspecific and perhaps evolutionary character^32,68^, which makes it recurrent between animals of the same family. Lévy patterns of movement have been reported for other taxonomic groups of mammals, such as primates^65^, and ungulates^66,69^, but this is the first analysis for marsupials.

A rigorous comparison of the *μ* values for all the different classes of data should be taken with some caution. In fact, even if the estimated standard error can seem relative small, this is only the error associated with the fitting, while other sources of uncertainty, originated by the data collection, the projecting procedure and the data selection, were not accounted for. These considerations are valid particularly for *M*. *paraguayana*, where few observations are available (see Table 2) and the final sample size is relatively small, a fact that suggests prudence. In fact, as the behaviour of the trajectories is generally heterogeneous, the average over different observations is important.

Taking into account these considerations, for the more robust case of *Philander* we can observe a slight decrease in the exponent value for non-oriented compared to oriented animals. This fact is consonant with more frequent long steps for oriented animals. Even so, the difference in the *μ* values is not significative enough to suggest a really different strategy of movement between oriented and non-oriented animals.

The *μ* values of both oriented and non-oriented individuals correspond to enhanced super-diffusion, approaching the ballistic limit, with no tendency towards random behaviours for non-oriented animals. The *μ* value is smaller than 1.4 for all the data-set, approaching the ballistic limit for the smaller exponents. Such small values have been previously identified in empirical studies for foraging albatrosses (*μ* = 1.75 for the average data set, but with individuals values as low as 1.14)^17^, for jellyfish (*μ* = 1.18)^70^ and marine predators (*μ* = 1.63)^40^.

Some of our estimated *μ* values are relatively close to the value *μ* = 4/3, which is found in the partial power-law behaviour present in 1-dimensional Continuous Correlated Random Walks, CCRW^15,38^. However, in this case, a clear and relevant exponential truncation must be present, which did not happen in our study. Also, the 95% confidence intervals for our *μ* values do not include the expected 4/3 value (see Table 2), but note that these estimations do not account for exponential truncation. The only exception would be the oriented *Philander frenatus*, (*μ* = 1.31 ± 0.02), but again only a very feeble exponential truncation can be suggested (see Figs 2 and 3). Our results could only be consistent with correlated random walks if the small arena precluded the detection of the real form of the exponential truncation, an aspect which should be object of future studies. Previous empirical studies exploring the connection between Lévy patterns and CCRW can be found in^15^ for the case of a small arthropod. Johnson^71^ reported that autocorrelation timescales of harbor seal and northern fur seal are several hours long, but Lévy walk movement patterns on these scales have not been measured. Lévy walk movement patterns with *μ* = 1.25 can be found in gray seals^12^.

The differences in the exponents of the truncated power-law models recorded in the two considered classes of behaviours are relatively weak. In contrast, a marked and relevant difference can be observed in the upper truncation value (*b*) of the Lévy walks, which for oriented animals is roughly twice the size of the not oriented case (see Table 2). This fact suggests that the difference in the movement strategy between the two behaviours can be found not in adjustments in the functional form that controls their walks, but in the length steps between turning points, which are in general considerably longer in the case of oriented animals. Therefore, animals could be adjusting the truncation scale of step lengths to switch between oriented and non-oriented movements. This phenomenon can be seen clearer looking at the mean number of reorientation points of a single walk. In the case of oriented animals, the number of reorientation points is roughly half that for not oriented animals. This is estimated considering that the number of reorientation points corresponds to the mean sample size of a single observation, in the approximation that all animals uses practically the entire length of the spool. From this spartan estimation, we obtain around 10 turning points for oriented and 20 for not oriented animals (see Table 2).

Lévy flights are considered to be the best solution for solving the problem of random search for sparsely distributed targets^25,41^. For this reason, the vast majority of empirical studies that reports Lévy processes are observations of foraging behaviour^11,15,40^. In contrast, the present study considers movements of animals that had been translocated to an unfamiliar site and different habitat^52^ (open areas in the matrix). Translocated animals generally try to return to familiar habitat and environment upon release, without foraging^72^. Forested areas are safe and familiar habitat for the three marsupials, while open areas represent unfavourable habitats for these animals where the predation risk by raptors, snakes and domestic dogs is high^6^. We note that even in the absence of predation events in the course of the experiments, animals may still show a behaviour typical of avoiding predation^7,73^. In fact, an unfamiliar environment induces animals to keep alert of potential predators and to find a safe place as soon as possible. Risk of predation generates a permanent stress, and takes a relevant part on the decision-making process^74^. For this reason, our empirical results seem to verify simulations of survival in patchy landscapes, which shows a generally decrease with *μ*^75^. Lévy walks are described outside of the context of optimal foraging only in a few cases, such as for shearwaters searching for their breeding colony after crossing vast regions of open ocean^76^ and for bacteria^77^.

Finally, an important difference from previous studies, is that, in our case, animals have no previous knowledge of the area of movement. For this reason, the detected movements are not the consequence of memory or of the familiarity with the specific release site. It is not plausible to completely rule out the possibility that the detected Lévy flights are the result of innate behaviour^78^, rather than an optimal strategy to search for safe patches under predation risk. The fact that Lévy patterns are related to some general innate behaviour, beyond the confines of optimal foraging, has gain interest in the literature^19^. The analysis of movements of *P*. *frenatus* released in similar conditions, but inside forest fragments, presented *μ* values comparable to the values estimated in the matrix (*μ* = 1.37 ± 0.02) (unpubl. data), supporting Lévy flights as result of innate behaviour.

To sum up, we describe by means of Lévy flights small-scale movements not associated with foraging, but with animals looking for a shelter in a risky habitat, where the memory of the area of movement can not play any role. The result is not dependent on the differences in the habits of the three species, and a weak differentiation between oriented and non-oriented animals is reported in the functional form of the truncated power-law behaviour.

## Supplementary information

Supplementary Information
