# Supplementary material for: Lévy like patterns in the small-scale movements of marsupials in an unfamiliar and risky environment
Source: Sci Rep. 2019 Feb 25;9:2737. doi: 10.1038/s41598-019-39045-0 (PMC6389917; doi:10.1038/s41598-019-39045-0)
Supplement: Supplementary file 1 — Supplementary Information [file 41598_2019_39045_MOESM1_ESM.pdf]

## SUPPLEMENTARY INFORMATIONS

# *Lévy like patterns in the small-scale movements of marsupials in an unfamiliar and risky environment*

*B. Ríos Uzeda, E. Brigatti and M. V. Vieira*

Table 1. Models selection between Pareto-truncated (PT), Pareto (PR) and Exponential (EX) distribution for the three species of marsupials. ΔAIC is the difference of the AIC values and wAIC is the weighted value of the AIC of the selected models.

| Species | Oriented | Model | Loglikelihood | AIC | ΔAIC | wAIC |
|---|---|---|---|---|---|---|
| *Didelphis aurita* | Yes | PT | -493 | 992 | 0 | 1 |
| | Yes | PR | -538 | 1080 | 88 | 0 |
| | Yes | EX | -616 | 1236 | 244 | 0 |
| | Not | PT | -1622 | 3249 | 0 | 1 |
| | Not | PR | -1829 | 3662 | 413 | 0 |
| | Not | EX | -1876 | 3755 | 506 | 0 |
| *Philander frenatus* | Yes | PT | -1262 | 2529 | 0 | 1 |
| | Yes | PR | -1362 | 2727 | 198 | 0 |
| | Yes | EX | -1759 | 3522 | 993 | 0 |
| | Not | PT | -2226 | 4458 | 0 | 1 |
| | Not | PR | -2474 | 4951 | 493 | 0 |
| | Not | EX | -2621 | 5246 | 788 | 0 |
| *Marmosa paraguayana* | Yes | PT | -111 | 227 | 0 | 1 |
| | Yes | PR | -126 | 255 | 28 | 0 |
| | Yes | EX | -230 | 465 | 238 | 0 |
| | Not | PT | -87 | 179 | 0 | 1 |
| | Not | PR | -106 | 215 | 36 | 0 |
| | Not | EX | -103 | 209 | 30 | 0 |

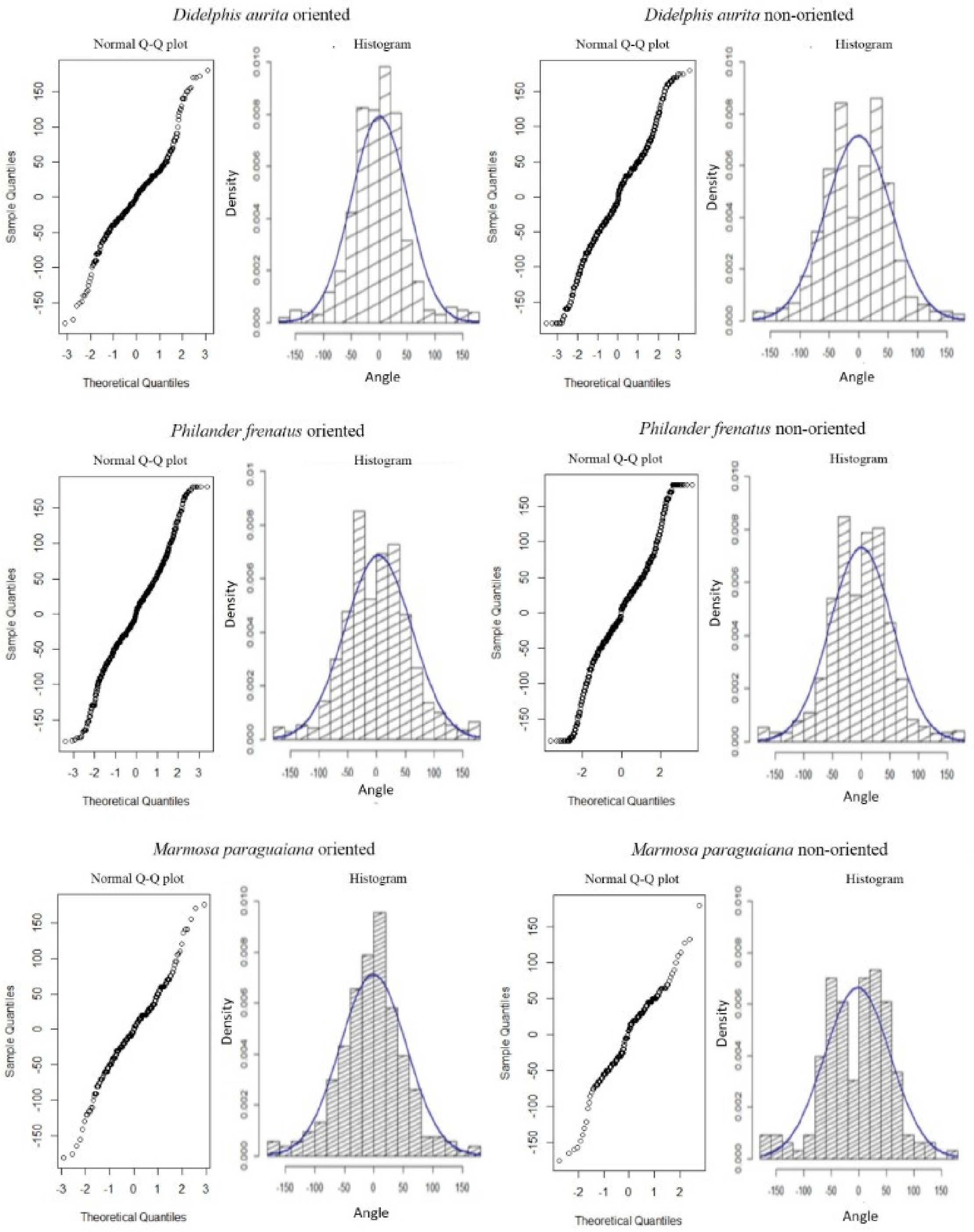


Figure 1. Distribution of angles (right) and normality test (left) for every species and for oriented and non-oriented animals. The histograms of turning angles are fitted by a normal distribution.
